# Application of a chemical reactivity database to predict toxicity for reactive mechanisms

**DOI:** 10.1186/1758-2946-3-S1-P18

**Published:** 2011-04-19

**Authors:** JAH Schwöbel, JC Madden, MTD Cronin

**Affiliations:** 1 School of Pharmacy and Chemistry, Liverpool John Moores University, Byrom Street, Liverpool, L3 3AF, UK

## 

Covalent binding of xenobiotic electrophiles to nucleophilic endogenous biomolecules, e.g. peptides or DNA, is a common molecular initiating event, leading to potentially irreversible toxic effects such as enhanced acute toxicity, skin sensitisation, or mutagenicity. This knowledge provides the basis for the *in silico* prediction of these toxicities. The potential for a chemical to be reactive can be determined experimentally by a number of chemical tests and therefore can be captured computationally to form (Q)SARs. Providing a source of in chemico data for the reactivity of electrophiles with reference nucleophiles could assist in the non-animal based risk assessment of chemicals for regulatory purposes and in the application of integrated testing strategies (ITS). For this reason, we have compiled a database from a full range of chemical reactivity assays containing various reactivity data of numerous electrophiles forming peptide and DNA adducts. This includes reactivity data, kinetic rate constants, and qualitative information regarding the adducts formed. The data collection facilitates the *in silico* profiling of toxicologically relevant compounds by grouping and category approaches, and allows for the combination of the following information: the identification of electrophilic compounds; their mechanistic applicability domain and compound class; physical-chemical properties; reactivity data; and toxicological data. These experimental reactivity data were linked to the computational prediction of reactive mechanisms for different modes of toxic action (e.g. acute aquatic toxicity), at which the results indicated that physically meaningful parameters are suitable to explain the varying behaviour of electrophiles. This could be applied for screening purposes based on structural information and the reactivity data could be used to elucidate mechanisms of toxic effects. The funding of the EU FP6 InSilicoTox Marie Curie Project (MTKD-CT-2006-42328) is gratefully acknowledged.

